# HESPER web - development and reliability evaluation of a web-based version of the humanitarian emergency settings perceived needs scale

**DOI:** 10.1186/s12889-020-8387-4

**Published:** 2020-03-12

**Authors:** K. Hugelius, M. Semrau, M. Holmefur

**Affiliations:** 1 School of Health Sciences, Örebro University, Örebro, Sweden; 2 Center for Global Health Research, Brighton and Sussex Medical School, Brighton, UK

**Keywords:** Needs assessment, Public health, Mental health, Humanitarian, Alternate forms reliability, Web based surveys

## Abstract

**Background:**

The Humanitarian Emergency Settings Perceived Needs Scale (HESPER) assesses a wide range of physical, psychological and social perceived needs across 26 questions, and can be used in humanitarian emergencies and disasters for needs assessment or research studies. The original HESPER collects data through individual interviews. Today, a large number of people have access to the internet, including in humanitarian emergencies and disasters. Therefore, this paper aimed to report the development, reliability evaluation and feasibility evaluation of the HESPER Web.

**Methods:**

First, the original HESPER was developed into a web based survey. Thereafter, alternate forms reliability between the HESPER and HESPER Web, and test-retest reliability for the HESPER Web, was evaluated using a study sample of 85 asylum seekers in Sweden in total.

**Results:**

The alternate forms reliability evaluation showed that the HESPER Web was a reliable instrument to assess perceived needs. Intraclass correlation coefficient (ICC) for total number of serious needs was 0.96 (CI 0.93–09.98, *p* < 0.001). Cohen’s κ was used to analyse the alternate forms reliability between the HESPER and HESPER Web item per item; the correspondence between HESPER and HESPER Web varied between 0.54 and 1.0 for the 26 questions. There was a strong nominal association in first priority need between the HESPER and HESPER Web (Cramer’s V 0.845, *p* < 0.001). In the test-retest reliability evaluation of HESPER Web, ICC was 0.98 (CI 0.97–0.99, *p* < 0.001), and Cohen’s κ varied between 0.53 and 1.0. There was a strong nominal association in first priority need between test and re-test (Cramer’s V 0.93, *p* < 0.001). The HESPER Web was experienced as easy and safe to use and was found less time consuming than the original HESPER interview, according to the study participants.

**Conclusion:**

The HESPER Web is a reliable and usable instrument to assess perceived needs. It can reduce a number of practical challenges both for needs assessment in disasters or humanitarian emergencies as well as in research.

## Background

Around 65 million people were affected by humanitarian emergencies and over 200 million by disasters in 2016 [1]. Despite these vast numbers of affected people, little is known on their perceived needs, which are needs as felt or experienced by affected people themselves. Needs assessment is fundamental in humanitarian relief, and also to understand health effects from such events in a public health perspective. The Humanitarian Emergency Settings Perceived Needs Scale (HESPER), developed by the World Health Organization (WHO) and King’s College London, aims to provide a quick, scientifically robust assessment of perceived needs of people affected by humanitarian emergencies or other disasters [2]. The instrument assesses a large number of physical, psychological and social needs across 26 items. The original version of the HESPER was designed to be used through face to face interviews, and the scale was psychometrically evaluated and considered valid and reliable to use in humanitarian contexts and disasters [3]. The HESPER is freely available from the WHO, and the manual as well as the scale itself is available in the original version in English and also in Arabic, Haitian Creole, French, Nepali, Spanish, and Urdu.

Today, almost 40% of the world population have access to the internet [4]. The proportion of people with access to the internet in humanitarian situations such as in refugee camps or other temporary living facilities is rising [5]. Access to internet is also considered as one of the most prioritized response interventions in sudden onset disasters [6]. Web based research methods have shown to reduce several methodological, practical, and ethical concerns in disaster research, such as challenges of timing, recruitment of participants, and the need for some kind of tracking mechanism to conduct traditional case-control, matched samples, or follow-up studies [7–9]. Web based data collections also offer less internal drop outs, less coding and processing errors, quicker data collections and analysis, and are often a more economical alternative to other types of surveys [10]. Therefore, a project consisting of several studies aimed to develop a self-administrated web based version of the HESPER, called HESPER Web, to evaluate the extent to which the web based version gives equivalent results as the interview version and the stability of those results, and to evaluate the feasibility of the HESPER Web in different contexts. This paper reports the development and first evaluations of HESPER Web in a sample of asylum seekers in Sweden.

## Methods

### Aim and design

The aim of this paper was to report on the development, reliability evaluation and usability evaluation of the HESPER Web.

The development and reliability evaluation of the HESPER Web was conducted across three phases; I) development of the HESPER Web, II) evaluation of alternate forms reliability, and III) test-retest reliability. In all three phases, the feasibility of the HESPER Web was also evaluated.

### Instrumentation

The original HESPER assesses needs as perceived by the people affected by the humanitarian emergency or disaster themselves, and consists of 26 questions covering physical, psychological and social needs. There is also possible to add context specific questions. Ratings are made by interviewers in a face-to-face interview with affected persons by defining whether or not each of the 26 questions are perceived by respondents to be a ‘serious problem’ (unmet need) or ‘no serious problem’ (no need). An additional open-ended question is also asked which gives the respondent the opportunity to identify any other serious needs not covered by the 26 core questions. At the end of the interview, respondents are then asked to rank their three most serious needs. A total sum score can be calculated by adding up the total number of ‘serious problem’ ratings. The HESPER includes as a minimum demographic questions on age, gender and current location. For this study, questions were added regarding country of origin, by providing the ten most common countries of origin for asylum seekers in Sweden together with a “stateless/ don’t want to say” alternative and a free text alternative. Also, questions on the usability and experience of using the HESPER Web were added, including technical problems, time to complete the survey, possibilities to answer the survey in privacy, potential experienced harm or other comments.

### Phase I; development of HESPER web

In the first phase, a web based version of the original HESPER was developed. The original HESPER survey phrasing in English was transformed into a web based survey tool called oru-survey, which is a protected survey tool and database for research purposes. The web based survey was created and posted as an Internet link using the www.abcde.xxx format. The survey could be accessed by both mobile phone, tablet and computer. In the web version, the exact phrasing from the HESPER English language version was used, using the whole question texts including explanations. The original HESPER ratings; “1: yes, a serious problem”, “0: No, no serious problem” or “9: Don’t know/ don’t want to say/ not applicable”, were used for each of the 26 questions, in accordance with the original scale. If the additional “other needs” question was ticked by study participants, there was a free text space to fill in, in order to explain the need further. For the ranking question, the survey was programmed to show only those items marked as 1 (“yes, serious problem”) and the study participant could mark one item as “most serious problem”, one as “second most serious problem” and another as “third most serious problem”.

To evaluate the phrasing as well as the web survey design, a panel of four senior researchers with expertise in psychology, public health and health statistics as well as ten international students with varied backgrounds (country of origin: Brazil, Egypt, India, Italy, Iran, Iraq, Japan, Mozambique, and Turkey) completed the survey and provided oral or written feedback to the research team on language, usability and design. The feedback was used throughout the developmental phase to refine the web survey.

### Phase II; alternate forms reliability evaluation

In the second phase, alternate forms reliability between the HESPER and HESPER Web was evaluated using a non-probability, voluntary study sample of newly arrived asylum seekers in Sweden. In total, approximately 250 adult asylum seekers were registered in the study region at the time of the data collection. Inclusion criteria were an age of 18 years old or more, ability to understand study information and survey questions in English, being an asylum seeker in Sweden and having access to a mobile phone (smart phone), tablet or computer. A power analysis indicated the need for 19 study persons in order to detect a statistically significant correlation and a power of 90%, based on the assumptions that the lowest acceptable Intra Class Correlation (ICC) was 0.7 and the target 0.9 [11]. Fifty-two study participants were recruited through information meetings in dedicated living areas for newly arrived asylum seekers and in social activity areas such as the Red Cross café in one region in Sweden. Data collection was conducted during the period of March 01 until May 31, 2018. The HESPER interviews were conducted in accordance with the HESPER manual [2] by the principal researcher (KH) and one assistant, in the study participants’ home or a private area in the social activity house. Half of the study sample were randomly selected to attend the face-to-face interview for the original HESPER first and then the HESPER Web next after approximately 1 week. The other participants answered the surveys in the opposite order. A reminder for the second data collection was sent to the study participant by text message (SMS) on day six, seven and eight after the first data collection. All data collection was done in English.

### Phase III; test-retest reliability evaluation

In the third phase, test-retest reliability for the HESPER Web was evaluated using another sample of asylum seekers in Sweden, but with the same inclusion criteria as above. The same power analysis was used for the alternate forms reliability evaluation and test-retest evaluation. Forty-four study participants were recruited through information meetings in social activity areas such as the Red Cross café in one region in Sweden. No study person was involved in both the alternate reliability evaluation sample and the test-retest evaluation sample. Data collection was conducted during the period of April 01 until June 24, 2018. Study participants completed the HESPER Web survey the first time in presence of a research assistant. A reminder to answer the web survey a second time was sent out by text message on day seven, eight and nine after the first data collection. All data collection was done in English.

### Data analyses phase II and III

Data from the web survey was automatically recorded into an Excel format, and thereafter imported into the statistical program SPSS (IBM Corp. Released 2016. IBM SPSS Statistics for Windows, Version 24.0. Armonk, NY: IBM Corp). All data were first checked for duplications, and dropouts were re-coded as missing data. For demographics and to analyse the evaluation questions, descriptive statistics was used. For the alternate forms reliability between the HESPER and HESPER Web, as well as for the test-retest validation of the HESPER Web, ICC, two-way mixed, absolute agreement [12] of total number of reported serious needs was calculated. To illustrate the agreement of mean number of needs between the HESPER and HESPER Web, test-retest Bland-Altman plots were constructed [13]. In the Bland-Altman plot, the difference between the two forms/tests was plotted against the mean score for each subject. The 95% limits of agreement were calculated as mean difference ± 1.95 SD. To assess agreement on an item level and precentral match between first priority need between the HESPER and HESPER Web as well as the results from the test-retest evaluation, Cohens ***κ*** was used. For calculation of association between the first prioritized need between HESPER and HESPER Web, and in the test-retest evaluation, Cramer’s V was calculated using a cross table methodology. Calculations were done by two of the researchers (KH and MH) and an external statistician.

### Ethical considerations

Permission to develop the HESPER Web was obtained from WHO. Ethical approval from the Regional Ethical Committee in Sweden (2017/481) was obtained for this study. In the study information, it was clearly described where study participants could turn to address any acute needs. Written informed consent was obtained from all study participants before interviews, and a digital informed consent was obtained before entering the survey questions in the web survey. All data collections were conducted in privacy, and information obtained by the data collectors was kept confidential. All data were stored at a protected research database server owned by Örebro University.

## Results

In total, 96 persons participated in phases II and III of the study, 52 in the alternate forms evaluation and 44 in the test-retest evaluation. Gender, age, country of origin and location at the time of the survey for all study participants, in all data collections, are shown in Table 1.

**Table 1:** Overview of study participant demographics for all data collections

		Alternate forms reliability evaluation		Test-retest evaluation	
		HESPER n (%)	HESPER Web n (%)	HESPER Web Data collection 1 n (%)	HESPER Web Data collection n (%)
**N**	96	52	41	44	32
	Drop outs	–	11 (21)	–	12 (27)
**Gender**	Male	28 (54)	22 (54)	23 (52)	17 (53)
	Female	24 (46.2)	19 (46)	21 (48)	15 (47)
**Age**	Mean (SD)	30.54 (8.0)	31.73 (8)	31.47 (9)	32.22 (11)
	Min	19	19	19	19
	Max	54	54	71	71
	*Missing*	*0*	*0*	*1*	*0*
**Location**	Location reported	51 (98.0)	41 (100)	28 (64)	24 (75)
	*Missing*	*1*	*0*	*16*	*8*
**Country of origin**	Afghanistan	15 (29)	13 (33)	3 (7)	3 (9)
	Eritrea	17 (33)	11 (28)	13 (30)	9 (28)
	Georgia	0 (0.0)	0 (0.0)	4 (9)	0 (0.0)
	Iran	3 (6)	1 (3)	1 (2)	0 (0.0)
	Iraq	1 (2)	1 (3)	2 (5)	2 (6)
	Somalia	5 (10)	5 (13)	7 (16)	6 (19)
	Sudan	1 (2)	1 (3)	1 (2)	1 (3)
	Syria	3 (6)	3 (8)	10 (23)	9 (28)
	Ukraine	1 (2)	1 (3)	0 (0.0)	0 (0.0)
	Stateless/Do not want to say	5 (10)	4 (10)	3 (7)	2 (6)
	*Missing*	*1*	*1*	*0*	*0*

### Alternate forms reliability evaluation between HESPER and HESPER web (phase II)

**Fig. 1:**
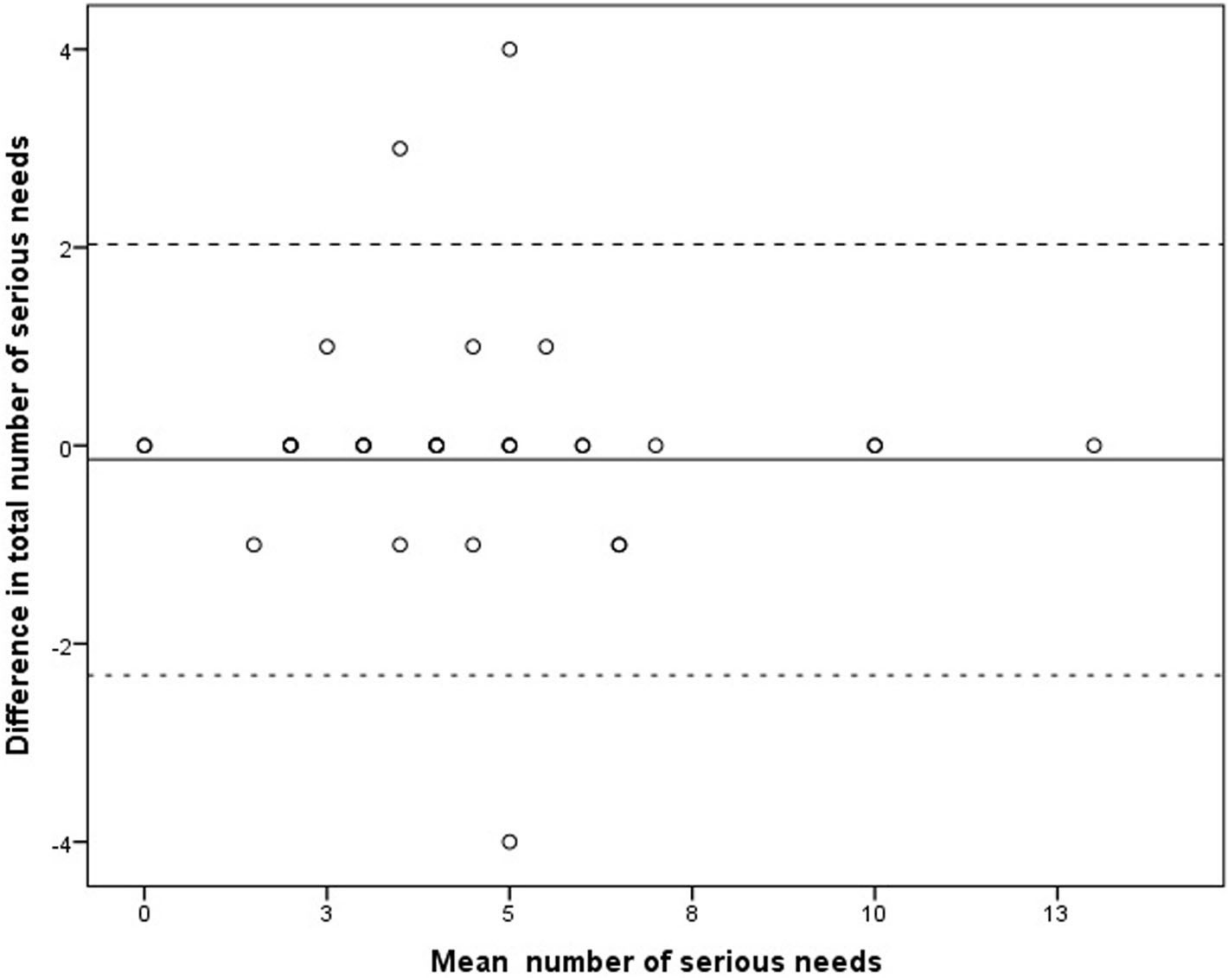
Bland Altman plot of the difference and mean number of serious needs between the HESPER and HESPER Web. Solid line illustrates the mean difference between HESPER and HESPER Web (− 0.146) and dotted lines illustrate the limits of agreement (− 2.32; 2.03)

Of the 52 study participants, 41 persons (79%) completed both the HESPER as an interview and the HESPER Web as a self-administrated web survey. The median between the two data collections was 6 days, with a range from 5 to 8 days. The mean number of reported total needs among the participants attending the HESPER interview was 3.93 (min 0, max 13, SD 2.81), and it was 4.07 (min 0, max 13, SD 2.80) for the persons completing the HESPER Web. There was no significant difference between the number of reported total needs between the HESPER and HESPER Web (mean − 0.15, SD 1.11, 95% CI -0.50; 0.20, *p* = 0.40). ICC for reported total needs was 0.96 (95% CI 0.93; 09.98, *p* < 0.001). A Bland Altman plot illustrated the measured difference of mean needs between the HESPER and HESPER Web (see Fig. 1). The agreement between the HESPER and HESPER Web for each of the 26 questions, calculated as Cohen’s *κ*, ranged from 0.543 to 1.0 (see Table 2).

**Table 2:** Number of participants reporting specific needs, and Cohen’s *κ* between HESPER and HESPER Web, per item

Item	HESPER	HESPER Web	Cohen’s ***κ***
	n (%)	n (%)	
Drinking water	0 (0.0)	0 (0)	–
Food	0 (0.0)	0 (0)	–
Place to live in	6 (15)	7 (17)	0.543
Toilets	0 (0)	0 (0)	–
Keeping clean	0 (0)	0 (0)	–
Clothes, shoes, bedding or blankets	0 (0)	0 (0)	–
Income or livelihood	24 (59)	26 (63)	0.796
Physical health	6 (15)	6 (15)	1.000
Health care	13 (32)	12 (29)	0.942
Distress	9 (22)	12 (29)	0.809
Safety	1 (2)	0 (0)	–
Education for your children	0 (0)	0 (0)	–
Care for family members	1 (3)	1 (3)	1.000
Support from others	9 (22)	10 (24)	0.795
Separation from family members	17 (43)	20 (50)	0.850
Being displaced from home	18 (44)	21 (51)	0.854
Information	7 (18)	5 (13)	0.805
The way aid is provided	8 (21)	8 (21)	1.000
Respect	9 (22)	8 (20)	0.926
Moving between places	14 (36)	12 (31)	0.885
Too much free time	10 (25)	12 (30)	0.875
Law and justice in your community	1 (3)	2 (6)	0.654
Safety or protection from violence for women in your community	0 (0)	0 (0)	–
Alcohol or drug use in your community	0 (0)	0 (0)	–
Mental illness in your community	0 (0)	0 (0)	–
Care for people in your community who are on their own	2 (7)	1 (3)	0.652
Other serious problems	5 (15)	4 (12)	0.872

The answers “no serious problem” and “not applicable/ don’t know” were grouped together as 0

For items with no reported needs, it was not possible to calculate Cohens ***κ***

When participants ranked their needs in order of priority, there was a strong nominal association in first priority need between the HESPER and HESPER Web (Cramer’s V 0.845, *p* < 0.000) (see Table 3). The precentral match between what items had been reported as the first priority need in the HESPER and HESPER Web was 81%.

**Table 3:**
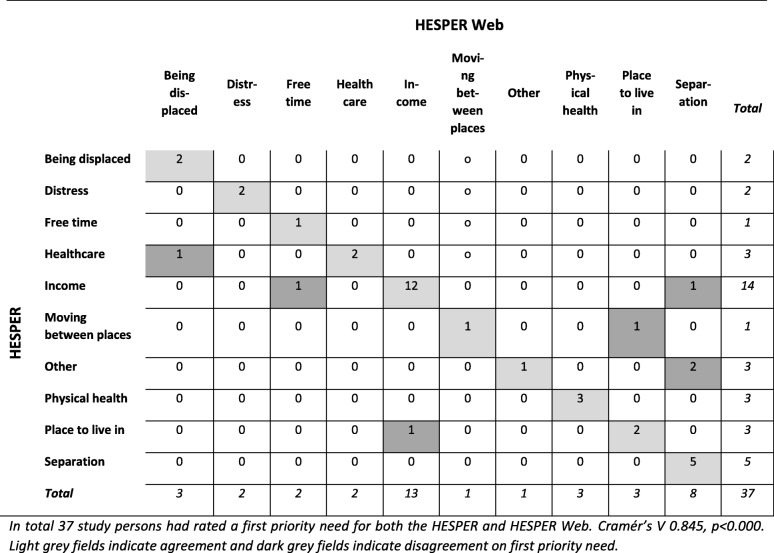
Correlation between first priority need in the HESPER and HESPER Web

In total 37 study persons had rated a first priority need for both the HESPER and HESPER Web. Cramér’s V 0.845, *p* < 0.000. Light grey fields indicate agreement and dark grey fields indicate disagreement on first priority need

### Test-retest reliability of HESPER web (phase III)

**Fig. 2:**
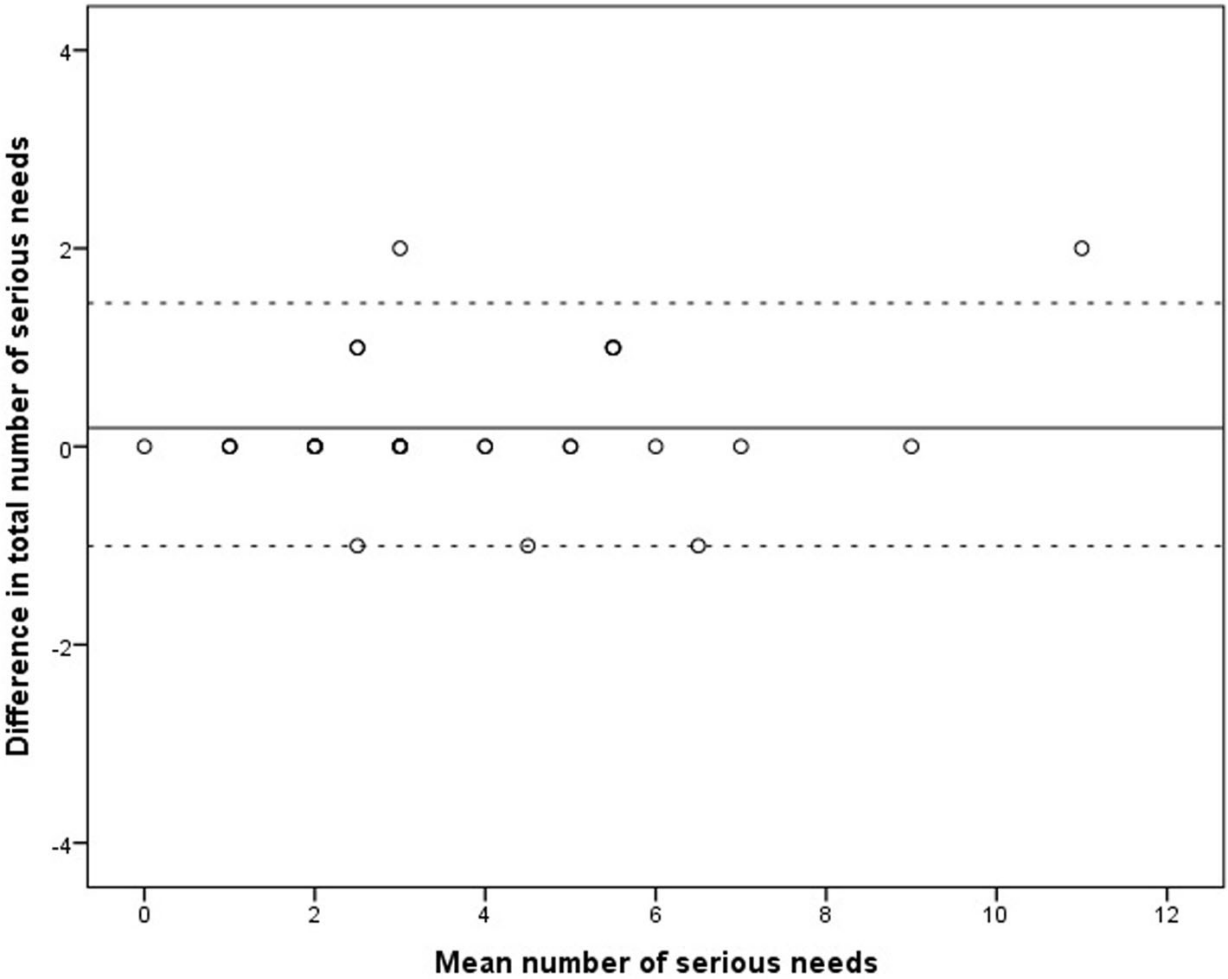
Bland Altman plot of the difference and mean number of serious needs between test and retest of HESPER Web. The solid line illustrates the mean difference between test and retest (0.188) and the dotted lines mark limits of agreement (− 1.07; 1.45)

Of the 44 study participants, 32 persons (73%) completed the HESPER Web twice, with a median of 8 days in between, ranging from 7 to 14 days. The mean number of reported total needs among participants answering the HESPER Web the first time was 4.03 (min 0, max 13, SD 2.66), and it was 3.84 (min 0, max 13, SD 2.50) the second time. There was no significant difference between in mean reported total needs between the first and second data collection (mean difference 0.19, SD 0.64, 95% CI -0.05; 0.42, *p* = 0.110). ICC for reported total needs was 0.984 (95% CI 0.97; 0.99, *p* < 0.001). A Bland Altman plot was calculated to show the measured difference of mean needs between the two data collections (se Fig. 2).

Cohen’s *κ* was used to analyse the test-retest reliability item per item for the HESPER Web (see Table 4). The agreement varied between 0.53 and 1.0. In the test re-test evaluation, 30 study participants reported a first priority need both times that they completed the HESPER Web survey. There was a strong nominal association in first priority need between test and re-test (Cramer’s V 0.930, *p* < 0.000) and a precentral match of 87% of the highest prioritized item.

**Table 4:** Number of participants reporting specific needs, and Cohen’s *κ* per item

Item	HESPER Web test	HESPER Web re-test	Cohen’s ***κ***
	n (%)	n (%)	
Drinking water	0 (0)	0 (0)	–
Food	0 (0)	0 (0)	–
Place to live in	7 (23)	7 (23)	1.000
Toilets	0 (0)	0 (0)	–
Keeping clean	0 (0)	0 (0)	–
Clothes, shoes, bedding or blankets	1 (3)	1 (3)	1.000
Income or livelihood	19 (59)	19 (59)	1.000
Physical health	6 (19)	6 (19)	1.000
Health care	9 (28)	9 (28)	1.000
Distress	12 (38)	12 (38)	1.000
Safety	1 (3)	0 (0)	–
Education for your children	0 (0)	0 (0)	–
Care for family members	1 (3)	1 (3)	1.000
Support from others	7 (22)	5 (16)	0.796
Separation from family members	12 (38)	12 (38)	1.000
Being displaced from home	12 (38)	12 (38)	1.000
Information	5 (16)	6 (19)	0.890
The way aid is provided	5 (16)	5 (16)	1.000
Respect	5 (16)	2 (6)	0.529
Moving between places	10 (31)	9 (28)	0.925
Too much free time	10 (31)	11 (34)	0.929
Law and justice in your community	2 (6)	2 (6)	1.000
Safety or protection from violence for women in your community	0 (0)	0 (0)	–
Alcohol or drug use in your community	0 (0)	0 (0)	–
Mental illness in your community	1 (3)	1 (3)	1.000
Care for people in your community who are on their own	1 (3)	0 (0)	–
Other serious problems	3 (11.1)	2 (7.4)	0.780

The answers “no serious problem” and “not applicable/ don’t know” were grouped together as 0

For items with no reported needs, it was not possible to calculate Cohens κ

### Drop outs

In the alternate form evaluation, 11 persons (21%) chose not to participate in the second data collection. All of them had undertaken the HESPER interview first, and were thereafter invited to the web based survey. In the test-retest evaluation, 12 persons (27%) did not answer the HESPER Web a second time. The internal drop out on an item level varied between 0 and 16 persons. The question with most missing data was the question on current location (16 missing answers, 36%).

### Usability evaluation of HESPER web

Most of the study participants used their own mobile phone to complete the HESPER Web (*n* = 65, 77%). Second most common was using someone else’s mobile phone (*n* = 8, 9%). Seven participants (8%) used a computer and two persons (2%) a tablet. To complete the HESPER Web took less time than the HESPER (see Table 5). The questions in the HESPER Web were found to be easy to understand, and only two out of 85 participants (2.4%) experienced technical problems or had problems answering the survey in private. Nobody stated that they had suffered harm from answering the HESPER Web (see Table 6).

**Table 5:** Time to complete the survey

	HESPER interview ***n*** = 52 n (%)	HESPER W ***n*** = 85 n (%)
**Less than five minutes**	24 (46)	79 (93)
**Six to ten minutes**	12 (23)	5 (6)
**11–15 min**	10 (19)	0 (0)
**More than 15 min**	2 (4)	0 (0)
**Missing data**	0 (0)	1 (1)

**Table 6:** Evaluation questions for HESPER Web

Total HESPER Web answers N = 85	Yes n (%)	No n (%)	Don’t know n (%)	Missing data n (%)
**Questions easy to understand**	80 (94)	0 (0)	4(5)	1(1)
**Experienced technical problems**	2 (2)	74 (87)	6 (7)	5 (6)
**Experienced harm from filling out the survey**	0 (0)	76 (89)	6 (7)	3 (4)
**Possible to answer the survey in private**	72 (85)	2 (2)	2(2)	9 (11)

## Discussion

The alternate forms and test-retest reliability evaluations showed that reliability for the HESPER Web was excellent to assess total number of needs, and strong in nominal association between first priority need. Most HESPER Web questions showed strong reliability on an item level in both the alternate forms reliability and test-retest reliability evaluations. The HESPER Web was experienced as easy and safe to use, and was found to be less time-consuming than the original HESPER interview.

Both the alternate forms and test-retest reliability coefficients were very high, despite the fact that the variance in the sample was somewhat low, i.e. few needs were reported by most participants, which tends to deflate the ICC [12]. The strong reliability (ICC 0.96) between the two alternate administration forms of the HESPER means that the HESPER Web gives equivalent information on the total number of perceived serious needs as the HESPER interview. The high reliability coefficient for the alternate forms should be viewed in light of the test-retest reliability results (ICC 0.98), which shows that a small fraction of the variance is due to just retesting, and the added variance from the alternate forms, i.e. the difference between the alternate forms and test-retest reliability coefficients, is minimal. The practical implications of these results are that the HESPER and HESPER Web can be used interchangeably and their results on total number of needs are equivalent. On an item level, some items did report a lower, but still moderate, correspondence between the HESPER and HESPER Web versions. As an example, distress was reported slightly more frequently in the HESPER Web than in the HESPER. Self-administered and web based instruments have been suggested to provide more genuine reporting of mental health issues by offering less experienced stigmatization than an interview or paper survey [9]. This supports the reliability of the results of the HESPER Web. The test-retest evaluation showed that the total number of reported needs was stable, which was also the case for most of the 26 items. The question regarding respect was the item that gained the lowest Cohens *κ*, and there were fewer reports on lack of respect as a serious problem during the second data collection. The reason for this is unknown, but it could be speculated that participating in the first round of data collection had the effect of lessening the burden of not feeling respected; however the limited number of participants reporting this specific need was small (five during the first data collection and two in the second) and so the difference simply could be due to chance. Some of the participants in this study were fluent English speakers and some were not, which may have influenced their interpretation of the questions. However, neither in the alternate reliability evaluation nor in the first round of the test-retest survey when research team staff were present, were there any obvious needs for language or other support by participants. Therefore, the original phrasing of the HESPER was used without changes in the HESPER Web survey.

The original HESPER is considered to be a valid and reliable instrument, and has been used in both needs assessments and for research purposes [2, 3, 14, 15]. Using a web based, self-administrated, instrument to assess perceived needs in humanitarian emergencies or disasters has several advantages. This study showed that the HESPER Web offers a quicker way to collect data on perceived needs than the original HESPER. As long as there is internet connection available, the HESPER Web enables a quick assessment and could also reach a large number of people in any phase of a humanitarian or disaster situation. The HESPER Web may offer better possibilities to reduce a common selection bias in disaster evaluations by enabling populations to participate that are rarely included in disaster research, such as people who move around, who have been evacuated from a disaster area or do not have access to a fixed location or mail address [16]. To use a self-administered instrument also reduces the costs and staff needed for individual interviews. In emergencies or disasters there might be practical challenges to physically reach the area, security concerns for the research team and/or the study participants or problems to ensure privacy for interviews. All these challenges may be reduced by using a self-administered web based instrument instead of face to face interviews [17].

However, to use a web based method for needs assessments or research purposes also has limitations. With interviews, the interviewer has the possibility to explain and clarify the questions if not understood, which might reduce the risk for drop outs. At the same time, the results from this study showed only minor drop outs, both internally and externally. The question with the highest level of missing data was the open question where study participants were given the opportunity to add any additional needs in their own words. Therefore, it can be recommended to identify given alternatives for all questions. When conducting HESPER interviews face by face, the interviewer does interact with the person, and may also provide specific advice or referrals if needed. When this option is limited using a self-administrated web based instrument such as the HESPER Web, it will therefore be of extra importance to inform the study participants where they should turn for support.

Not all study populations or contexts are suitable for web-based needs assessment. There are several reasons for this, including limited access to the internet or means for answering the survey, limited privacy when answering the survey, illiteracy, access to smartphone or computer or severely traumatized populations where personal contact is necessary to provide immediate support [17]. The responsibility to use a valid and proper instrument and data collection procedure, considering the context and study population, is always with the researchers or head of organization, and not the affected population. At the same time, disaster-related research is often well tolerated by the study population, and not conducting needs assessments or research studies in emergencies or disasters may also be unethical [18, 19]. When using web based surveys including personal data, such as data on perceived needs, it must be assured that storing of data is adequately secured. Especially in humanitarian contexts and conflict areas, protection of data and personal information is extremely important [20] and must be secured by the investigator. In this study, no personal identifying information such as name or identification number was used in the surveys. In order to protect individuals completing the survey, our recommendation is to use the HESPER Web without collecting information on IP address, name, id number, physical address or other information that could identify the study participant, and to store data in a secure way [20]. To summarize, HESPER Web can enable new possibilities to gain data for research and in humanitarian response assessments. By using a self-administrated web survey, several practical challenges common in disaster-related research can be reduced, and the HESPER Web offers a quick and scientifically robust way of collecting a large number of data also in populations or locations where personal access might be limited. In addition, the instrument offers new possibilities to include populations that have not been addressed before, as long as they have internet access.

The context for this study was newly arrived asylum seekers, located in a high-income country with excellent infrastructure and non-crises environment. Using such a sample to evaluate an instrument developed for low- and middle-income countries settings might be questioned. The reason for the choice of this study area was to ensure a proper psychometric evaluation of the instrument, which was the main focus for this study. Asylum seekers also in high-income countries have been shown to be a vulnerable population reporting significant more health problems than others [21] and were therefore considered to be a relevant population for the psychometric evaluation. Using a voluntary, non-randomized probability sample means that the generalization of the needs reported has to made with caution. A stratified or quota convenience sampling was not considered because of the need for specific language skills among the study participants as well as limited access to detailed registers supporting such sampling. However, this paper aimed to report on the development and reliability of the HESPER Web. The actual needs reported will be analyzed, presented and discussed elsewhere.

In order to further evaluate the feasibility and practical use of the HESPER Web, studies on its use in other humanitarian contexts and both in low- middle and high-income countries is needed and will be carried out in the nearby future. The practical and scientific use of a voluntary study sample instead of a randomized or cluster sample of study participants in humanitarian contexts also needs to be evaluated. To map the availability of internet access in both long term humanitarian contexts and sudden onset disasters is also a question of importance to estimate the representativeness of data collected with the HESPER Web. Also, the use of HESPER Web to measure perceived needs among specific populations, such as migrants, minorities or vulnerable populations, and the practical use of HESPER Web might be of interest for future public health studies.

## Conclusion

The HESPER Web is a reliable and usable tool to assess perceived needs. It can reduce a number of practical challenges both for needs assessment in disasters or humanitarian emergencies as well as in research.

## Data Availability

The survey tool web link, and data sets used during the current study are available from the corresponding author on reasonable request. The original HESPER scale is freely available from the World Health Organization (WHO) at: https://www.who.int/mental_health/publications/hesper_manual/en/. The HESPER Web will be available from WHO after the research project is finalized. The WHO will own the legal rights to the HESPER Web.

## Abbreviations

HESPER: The Humanitarian Emergency Settings Perceived Needs Scale
ICC: Intra Class Correlation
WHO: World Health Organization
